# Association of liver elastography measurements with poor glycaemic control in elderly patients with type 2 diabetes

**DOI:** 10.20945/2359-4292-2025-0034

**Published:** 2025-10-28

**Authors:** Qian Zhang, Li Cao, Yu-Min Wang, Xue-Song Wang, Xia Cao, Pan Li, Yi-Min Wang, Xiang-Dong Hu, Xian-Quan Shi

**Affiliations:** 1 Department of Ultrasound Medicine, Beijing Friendship Hospital, Capital Medical University, Xicheng District, Beijing; 2 Department of General Medicine, Baizhifang Community Health Service Center, Xicheng District, Beijing

**Keywords:** Type 2 diabetes mellitus, liver elastography, glycaemic control, elderly patients, continuous glucose monitoring

## Abstract

**Objective:**

The relationship between liver health and glycaemic control in elderly patients with diabetes remains poorly understood. In this study, the value of liver elastography in identifying associations with poor glycaemic control among elderly patients with type 2 diabetes mellitus was investigated.

**Subjects and methods:**

In total, 90 elderly patients (aged ≥ 60 years) with type 2 diabetes mellitus were enrolled in this prospective observational study. All participants underwent liver elastography using FibroScan^®^ and continuous glucose monitoring (CGM). Liver stiffness measurements (LSMs) and the controlled attenuation parameter (CAP) were obtained. Glycaemic control was assessed through multiple parameters, including the time in range (TIR), time above range (TAR), glycaemic variability, and mean glucose levels. Poor glycaemic control was defined as a TIR < 70%. The mean age of the participants was 64.0 ± 10.5 years, with 65.6% being female. The mean liver stiffness was 6.1 ± 7.8 kPa, and the mean CAP was 266.0 ± 54.7 dB/m.

**Results:**

Patients with higher liver stiffness (>8.0 kPa) had a significantly lower TIR (68.7% versus 83.5%, p<0.001) than those with normal liver stiffness (<5.5 kPa). LSMs were strongly negatively correlated with the TIR (r = -0.42, p < 0.001) and positively correlated with the mean glucose level (r = 0.38, p < 0.001). Multivariate analysis revealed that increased liver stiffness was independently associated with poor glycaemic control (adjusted OR = 1.28, 95% CI: 1.14-1.44; p < 0.001).

**Conclusion:**

ROC analysis revealed an exploratory LSM cut-off value of 6.8 kPa for association with poor glycaemic control (AUC = 0.76; sensitivity = 71.2%; specificity = 78.9%). LSMs via transient elastography are independently associated with poor glycaemic control in elderly patients with type 2 diabetes. An LSM threshold of 6.8 kPa may help identify patients who are more likely to present with poor glycaemic control.

## INTRODUCTION

Type 2 diabetes mellitus (T2DM) represents a significant global health challenge, particularly among the elderly population, with its prevalence increasing dramatically over the past decades (^1^). The management of diabetes in elderly patients presents unique challenges due to multiple comorbidities, altered metabolism, and complex pathophysiological changes associated with ageing (^2^). Recent evidence suggests a strong bidirectional relationship between liver health and glycaemic control, with liver dysfunction potentially playing a crucial role in difficulties managing diabetes (^3^).

The liver serves as a central metabolic hub in glucose homeostasis, and its structural and functional integrity is essential for maintaining optimal blood glucose levels. Recent studies have demonstrated that early-stage liver fibrosis, even before clinical manifestation, may significantly impact glucose metabolism and insulin resistance (^4^). This relationship becomes particularly relevant in elderly patients with diabetes, where age-related changes in liver function may compound the challenges of glycaemic control (^5^).

Liver elastography, particularly transient elastography (TE), has emerged as a noninvasive, reliable method for assessing liver stiffness and detecting early-stage fibrosis (^6^). Its advantages include reproducibility, ease of use, and immediate results, making it particularly suitable for elderly patients who may be poor candidates for invasive liver biopsies (^7^). While liver elastography is traditionally used in viral hepatitis and fatty liver disease assessment, emerging evidence suggests its potential utility in diabetes management (^8^).

The use of liver elastography as a tool for identifying associations with poor glycaemic control represents a novel approach in diabetes management. Recent studies have shown correlations between increased liver stiffness measurements and poor glycaemic control in various patient populations (^9^). However, the specific application of this technology in elderly patients with diabetes, particularly in identifying those with management challenges, remains underexplored (^10^).

Understanding the relationship between liver elasticity and glycaemic control could provide valuable insights into the pathophysiological mechanisms underlying difficult-to-manage diabetes in elderly patients. Early detection of liver fibrosis through elastography might help identify patients at risk of poor glycaemic control, allowing for more targeted and effective interventions (^11^). Furthermore, this approach could help explain why some elderly patients with diabetes experience persistent hyperglycaemia despite adhering to standard treatment protocols (^12^).

Research has indicated that hepatic insulin resistance, which is often associated with liver fibrosis, can significantly impact diabetes management (^13^). The role of the liver in glucose metabolism, including glycogen storage and gluconeogenesis, makes it a critical organ for maintaining glycaemic homeostasis. Recent studies have suggested that even subtle changes in liver structure, as detected by elastography, might precede clinically significant alterations in glucose metabolism (^14^).

The importance of early identification of factors contributing to poor glycaemic control cannot be overstated, particularly in the elderly population, where diabetes complications can have severe consequences (^15^). Traditional methods for assessing difficulties in diabetes management often focus on clinical parameters such as HbA1c levels, medication compliance, and lifestyle factors. However, these approaches may not fully capture the underlying physiological barriers to achieving optimal glycaemic control (^16^).

Therefore, this study aims to investigate the value of liver elastography in identifying associations with poor glycaemic control among elderly patients with type 2 diabetes, with a particular focus on identifying early-stage liver fibrosis as a potential marker of challenging diabetes management.

## SUBJECTS AND METHODS

### Study design and population

This prospective observational study was conducted at the Department of Endocrinology and Geriatrics of our medical centre between May 2024 and July 2024. The study protocol was approved by the Institutional Review Board of Beijing Hui Muslim Hospital (Approval number: 20230043), and written informed consent was obtained from all participants. The study was conducted in accordance with the Declaration of Helsinki. A convenient sample of consecutive outpatients who met the inclusion criteria was used in the present study.

The inclusion criteria were as follows: (^1^) patients aged 60 years or older with previously diagnosed type 2 diabetes mellitus; (^2^) regular follow-up at our diabetes clinic for at least 6 months; (^3^) complete medical records, including continuous glucose monitoring (CGM) data and glycated haemoglobin (HbA1c) measurements; and (^4^) the ability to provide informed consent and comply with the study procedures. The exclusion criteria were (^1^) a history of viral hepatitis, autoimmune hepatitis, or other known liver diseases; (^2^) alcohol consumption exceeding 20 g per day; (^3^) the use of medications known to affect liver function within the past 6 months; (^4^) the presence of hepatic tumours or severe cardiovascular diseases; and (^5^) the inability to complete liver elastography examination.

### Clinical and laboratory assessment

Comprehensive clinical evaluations were performed for all participants at baseline. Demographic data, including age, sex, duration of diabetes, current medications, and comorbidities, were collected through standardized questionnaires and medical record review. Anthropometric measurements, including height, weight, body mass index (BMI), and waist circumference, were obtained using calibrated instruments following standardized protocols.

Blood samples were collected after an overnight fast of at least 8 hours. Laboratory assessments included fasting plasma glucose, HbA1c, liver function tests (ALT, AST, GGT, and ALP), lipid profiles (total cholesterol, triglycerides, HDL-C, and LDL-C), and other relevant biochemical parameters. All laboratory tests were performed in our hospital’s clinical laboratory using standardized methods with regular quality control procedures.

### Data collection

The medication classes extended biochemical panels and waist circumferences were extracted and are summarized in **Supplementary Table 1**. These included oral antidiabetic medications, GLP-1 receptor agonists, SGLT2 inhibitors, insulin therapy regimens, and other relevant medications. Comprehensive biochemical parameters, including inflammatory markers (C-reactive protein), were also documented.

### Liver elastography assessment

Liver elastography measurements were performed using FibroScan^®^ (Echosens, Paris, France) by experienced technicians who had performed at least 500 examinations prior to the study. All measurements were conducted following a standardized protocol, with patients in the supine position with the right arm in maximum abduction. The M probe was used as the default, while the XL probe was employed for patients with a BMI ≥ 30 kg/m² or when indicated by the device’s automatic probe selection tool.

Transient elastography (TE) measurements were performed in the right liver lobe through intercostal spaces while patients held their breath for 5-10 seconds. Ten valid measurements were obtained for each patient, and the median value was used for analysis. Examinations were considered reliable when the following criteria were met: at least 10 valid measurements, an interquartile range (IQR) to median ratio of ≤ 30%, and a success rate of ≥ 60%. The liver stiffness measurement (LSM) results were expressed in kilopascals (kPa). Cut-offs were chosen according to Eddowes et al. (2019) for NAFLD (≥5.5 kPa for early fibrosis) and the ADA 2025 consensus (≥8 kPa, which is suggestive of significant fibrosis) (^17,18^).

### Glycaemic control assessment

Glycaemic control was evaluated through multiple parameters to ensure a comprehensive assessment. All participants underwent professional continuous glucose monitoring (CGM) using the Dexcom G6^®^ system for a minimum period of 14 days. CGM data were analysed for various metrics, including mean glucose level, glucose management indicator (GMI), time in range (TIR, 3.9-10.0 mmol/L), time above range (TAR, > 10.0 mmol/L), time below range (TBR, < 3.9 mmol/L), and glycaemic variability measures, such as the coefficient of variation (CV) and standard deviation (SD).

HbA1c measurements were obtained at baseline and during follow-up visits using high-performance liquid chromatography (HPLC). Poor glycaemic control was defined based on a composite assessment including an HbA1c > 7.5% (58 mmol/mol) despite optimal medical therapy, significant glycaemic variability (CV > 36%), or a TIR < 70%, according to international consensus guidelines.

### Statistical analysis

Statistical analyses were performed using SPSS version 26.0 (IBM Corp., Armonk, NY, USA). The normality of continuous variables was assessed using the Shapiro-Wilk test. Continuous variables are expressed as the mean ± standard deviation or median (interquartile range), as appropriate. Categorical variables are presented as numbers and percentages. Comparisons between groups were performed using Student’s t test or the Mann-Whitney U test for continuous variables and the chi-square test or Fisher’s exact test for categorical variables. Correlations between liver stiffness measurements and glycaemic parameters were assessed using Pearson’s or Spearman’s correlation coefficients as appropriate.

Multivariate logistic regression analysis was performed to identify independent associations with poor glycaemic control, with adjustment for potential confounding factors, including age, sex, BMI, diabetes duration, and medication use. The exploratory value of liver stiffness for identifying associations with poor glycaemic control was assessed using receiver operating characteristic (ROC) curve analysis, with calculation of the area under the curve (AUC), sensitivity, and specificity. ROC analysis was exploratory and aimed to illustrate the strength of the association rather than to develop a diagnostic algorithm. A two-sided p value < 0.05 was considered to indicate statistical significance. Sample size calculation was performed on the basis of preliminary data, indicating that 85 patients would provide 80% power to detect a significant correlation between liver stiffness and glycaemic control parameters, with an α level of 0.05.

## RESULTS

### Baseline characteristics

A total of 90 elderly patients with type 2 diabetes mellitus were enrolled in the study. The mean age was 64.0 ± 10.5 years, with women comprising 65.6% (59/90) of the study population. The baseline demographic and clinical characteristics are presented in **Table 1**. Additional medication use patterns, extended biochemical parameters, including HbA1c levels, and anthropometric measurements, including waist circumference, are detailed in **Supplementary Table 1**.

### Liver elastography findings

Liver stiffness measurements (LSMs) were successfully obtained for all participants, with a median value of 4.6 kPa (interquartile range: 3.9-5.9 kPa). On the basis of established cut-off values, participants were stratified into three groups: normal liver stiffness (<5.5 kPa, n = 58), mildly increased stiffness (5.5-8.0 kPa, n = 24), and significantly increased stiffness (>8.0 kPa, n = 8). The controlled attenuation parameter (CAP) measurements revealed a mean value of 266.0 ± 54.7 dB/m, suggesting varying degrees of hepatic steatosis among the study population.

### Glycaemic control parameters and their relationships with liver stiffness

Analysis of continuous glucose monitoring data revealed significant variations in glycaemic control across different liver stiffness groups. The mean time in range (TIR) was 79.2%, with notable differences among the liver stiffness groups (**Table 2**). The mean HbA1c was 7.8 ± 1.2%, which correlated moderately with the TIR (r = -0.36, p < 0.001), supporting the consistency between CGM and traditional glycaemic markers.

### Correlation analysis

Significant correlations were observed between liver stiffness measurements and various glycaemic control parameters. Liver stiffness was strongly negatively correlated with the TIR (r = -0.42; p < 0.001) and positively correlated with the mean glucose level (r = 0.38; p < 0.001). Additionally, the CAP values were moderately correlated with the glycaemic variability measures (r = 0.32; p = 0.002). Waist circumference was moderately correlated with the LSM (r = 0.28; p = 0.007), as detailed in **Supplementary Table 1**.

### Multivariate analysis

Multivariate logistic regression analysis revealed that after adjusting for age, sex, BMI, and diabetes duration, increased liver stiffness remained independently associated with poor glycaemic control (defined as a TIR < 70%). The adjusted odds ratio for poor glycaemic control was 1.28 (95% CI: 1.14-1.44; p < 0.001) for each 1 kPa increase in liver stiffness (**Table 3**).

### Exploratory value of liver elastography

ROC curve analysis revealed that liver stiffness measurements showed good exploratory value for identifying patients associated with poor glycaemic control, with an area under the curve (AUC) of 0.76 (95% CI: 0.68-0.84). The optimal cut-off value was determined to be 6.8 kPa, with a sensitivity of 71.2% and a specificity of 78.9% for association with poor glycaemic control (**Figure 1**).

## DISCUSSION

This study revealed a significant association between liver stiffness measurements and poor glycaemic control in elderly patients with type 2 diabetes mellitus. Our findings reveal that increased liver stiffness, as measured by transient elastography, is strongly correlated with poor glycaemic control parameters and may serve as an early indicator of potential challenges in diabetes management in this population.

The observed relationship between liver stiffness and glycaemic control parameters provides important insights into the pathophysiological connections between hepatic health and diabetes management. Our results revealed that patients with higher liver stiffness measurements (>8.0 kPa) demonstrated a significantly lower TIR (68.7%) than those with normal liver stiffness (83.5%). This finding aligns with recent research indicating that early-stage liver fibrosis may impair hepatic glucose metabolism and insulin sensitivity (^19^). The crucial role of the liver in maintaining glucose homeostasis through glycogen storage and gluconeogenesis makes it particularly relevant in diabetes management, and our findings suggest that even subtle changes in liver structure may have meaningful impacts on glycaemic control.

The strong negative correlation between liver stiffness and the TIR (r = -0.42; p < 0.001) observed in our study extends previous research that focused primarily on HbA1c as the sole indicator of glycaemic control (^20^). This more comprehensive evaluation of glycaemic parameters through continuous glucose monitoring provides a deeper understanding of the relationship between liver health and daily glucose fluctuations. The association between increased liver stiffness and higher glycaemic variability (CV) suggests that liver dysfunction may contribute to unstable glucose levels, a finding particularly relevant for elderly patients who are more vulnerable to glycaemic excursions (^21^).

Our multivariate analysis revealed that liver stiffness remained independently associated with poor glycaemic control even after adjusting for traditional risk factors. This finding is particularly noteworthy, as it suggests that liver elastography might provide additional prognostic information beyond conventional clinical parameters (^22^). The adjusted odds ratio of 1.28 for each 1 kPa increase in liver stiffness aligns with recent studies showing the incremental value of liver health assessment in diabetes management (^23^).

The exploratory cut-off value of 6.8 kPa identified in our study demonstrates good associative accuracy for poor glycaemic control, with reasonable sensitivity and specificity. This threshold is lower than the traditional cut-off values used for diagnosing significant liver fibrosis, suggesting that even mild increases in liver stiffness may impact glucose metabolism (^24^). This finding has important clinical implications, as it indicates that liver health assessment might be valuable earlier in the course of diabetes management than previously recognized. Our findings suggest that LSM may help identify patients who are more likely to present with poor glycaemic control, although it should not be used as a standalone diagnostic tool (^25^).

Our ROC-derived cut-off of 6.8 kPa should be regarded as a sensitivity-oriented screening threshold: for any patient exceeding this threshold, second-tier noninvasive tests or hepatology referrals may be necessary to determine MASLD severity. The current ADA consensus recommends action at ≥ 8 kPa, a level that is enriched for histologic ≥ F3 fibrosis (^17^), while large multicentre NAFLD cohorts report optimal thresholds of 8.2-9.7 kPa for ≥ F2-F3 fibrosis (^18^). A slightly lower value of 6.8 kPa therefore prioritises sensitivity and must not be used as a stand-alone diagnostic boundary.

The mean CAP value of 266.0 ± 54.7 dB/m in our cohort indicates the presence of varying degrees of hepatic steatosis, a finding consistent with the known association between diabetes and fatty liver disease (^26^). The moderate correlation between CAP values and glycaemic variability measures suggests that hepatic fat content may also play a role in glucose regulation, potentially through mechanisms involving insulin resistance and altered hepatic glucose production (^27^).

The sex distribution in our study, which including more female patients (65.6%), reflects the demographic characteristics of elderly patients with diabetes in many Asian countries (^28^). The observed relationships between liver stiffness and glycaemic control remained consistent across sexes, suggesting that the associative value of liver elastography may be applicable regardless of sex.

The use of continuous glucose monitoring in our study provides a more comprehensive assessment of glycaemic control compared to traditional metrics. The finding that increased liver stiffness is correlated with both reduced time in range and increased glycaemic variability supports the concept that liver dysfunction may affect multiple aspects of glucose homeostasis (^29^). The consistency between the HbA1c and CGM parameters (r = -0.36) further validates our findings and strengthens their clinical applicability.

Our results have several clinical implications. First, they suggest that liver elastography could be a valuable tool for risk stratification in elderly patients with diabetes, potentially identifying those who may require more intensive monitoring and management strategies (^30^). Second, the finding that even mild increases in liver stiffness correlate with poor glycaemic control suggests that early attention to liver health might be important in optimizing diabetes management (^31^).

The relationship between liver stiffness and glycaemic control appears to be particularly relevant in elderly patients, who may have accumulated metabolic changes over time. The observed associations suggest that age-related changes in liver structure might contribute to the increased difficulty in achieving optimal glycaemic control in this population (^32^). This understanding could help explain why some elderly patients experience persistent hyperglycaemia despite adequate medication adherence and lifestyle modifications.

### Study limitations

This study has several limitations that should be acknowledged. First, this was a single-centre study with a moderate sample size, which may limit the generalizability of our findings. The convenience sampling method used in this study may have contributed to the observed predominance of female participants (65.6%) in our cohort, which could affect the external validity of our results. Second, the cross-sectional design limits our ability to establish causal relationships between liver stiffness and glycaemic control. Third, while we adjusted for multiple confounders, residual confounding from unmeasured variables cannot be excluded. Finally, our findings require validation in larger, multicentre cohorts with more diverse populations before widespread clinical implementation can be recommended.

In conclusion, this study demonstrates that liver stiffness measurement by transient elastography is independently associated with poor glycaemic control in elderly patients with type 2 diabetes mellitus. The identified threshold of 6.8 kPa provides a practical reference point for identifying patients who are more likely to present with poor glycaemic control. These findings suggest that routine assessment of liver elastography could be a valuable addition to the comprehensive evaluation of elderly patients with diabetes, particularly in identifying and understanding challenges in glucose management.

## Data Availability

the datasets used or analyzed during the current study are available from the corresponding author on reasonable request.
